# Identification of lead chemotherapeutic agents from medicinal plants against blood flukes and whipworms

**DOI:** 10.1038/srep32101

**Published:** 2016-08-30

**Authors:** Phurpa Wangchuk, Paul R. Giacomin, Mark S. Pearson, Michael J. Smout, Alex Loukas

**Affiliations:** 1 Centre for Biodiscovery and Molecular Development of Therapeutics, Australian Institute of Tropical Health and Medicine, James Cook University, Cairns, QLD, Australia

## Abstract

Schistosomiasis and trichuriasis are two of the most common neglected tropical diseases (NTD) that affect almost a billion people worldwide. There is only a limited number of effective drugs to combat these NTD. Medicinal plants are a viable source of parasiticides. In this study, we have investigated six of the 19 phytochemicals isolated from two Bhutanese medicinal plants, *Corydalis crispa* and *Pleurospermum amabile*, for their anthelmintic properties. We used the xWORM technique and Scanning Electron Microscope-based imaging to determine the activity of the compounds. Of the six compounds tested, isomyristicin and bergapten showed significant anthelmintic activity against *Schistosoma mansoni* and *Trichuris muris* with bergapten being the most efficacious compound one against both parasites (*S. mansoni* IC_50_ = 8.6 μg/mL and *T. muris* IC_50_ = 10.6 μg/mL) and also against the schistosomulum stage of *S. mansoni*. These two compounds induced tegumental damage to *S. mansoni* and affected the cuticle, bacillary bands and bacillary glands of *T. muris*. The efficacy against multiple phylogenetically distinct parasites and different life stages, especially the schistosomulum where praziquantel is ineffective, makes isomyristicin and bergapten novel scaffolds for broad-spectrum anthelmintic drug development that could be used for the control of helminths infecting humans and animals.

Schistosomiasis and trichuriasis are major contributors to the disease burden in both humans and domestic animals, affecting the social and economic progress of many developing countries^1^,^2^,^3^. There are no vaccines, and only a handful of anthelmintic drugs exist to treat these infections. The development of drug-resistant helminths of livestock has been reported, and there is a looming threat of resistance for the few human drugs that are currently used in mass drug administration programs in developing countries^4^,^5^,^6^. This has necessitated the discovery of lead compounds and development of new anthelmintic drugs.

Natural products, especially medicinal plants, have a bewildering diversity of allelochemicals with unusual structures and have been a reliable source of chemotherapeutic moieties^7^, including for anthelmintic drug discovery. For example, artemisinin and its derivatives (artemether) isolated from the Chinese medicinal plant, *Artemesia annua*, have a unique trioxane structure that is an essential frontline antimalarial drug which also has anti-schistosome and anti-cancer properties^8^,^9^. Another new antimalarial drug lead candidate, simplicifolianine, has been discovered from *Meconopsis simplicifolia*^10^. Despite their proven reputation as a source of antimalarials, plants have been relatively under-explored as a source of anthelmintic compounds. Athanasiadou *et al.*^11^ described a number of plants with anthelmintic properties under controlled experimentation, either through feeding of whole plant or plant extracts to parasitised hosts. While some plant extracts have strong bioactivities, even against drug-resistant worms, the use of crude extracts is fraught with complexity in terms of dose standardisation and mechanism of drug action. Crude plant extracts contain mixtures of allelochemicals, which may have synergistic, antagonistic or even superimposed bioactivities. For this reason, isolating, quantifying and identifying the bioactive compounds in plant extracts is a prerequisite for drug discovery and will accelerate the development of new generations of anthelmintic compounds.

Drawing inspiration from Bhutanese traditional medicine (BTM) we explored the anthelmintic properties of two medicinal plants – *Corydalis crispa* and *Pleurospermum amabile,* against *S. mansoni* and *T. muris*. These plants have been traditionally used in BTM as cleansing and detoxification agents, and also in treating liver disease, dyspepsia and other gastrointestinal infections that bear relevance to parasitic diseases^12^. The bitter root of *C. sempervirens*, which is a close relative of *C. crispa*, is also used as an anthelmintic and emmenagogue^13^. Ecologically, these plants have been observed to avoid attack from insects, indicative of their anti-feedant properties. The crude extracts and a small number of isolated compounds from *C. crispa* and *P. amabile* have been reported to have anti-parasitic activities against *Trypanosoma brucei rhodesiense*^12^ and the multi-drug resistant strain (K1CB1) of *Plasmodium falciparum*^14^,^15^. However, none of the 19 compounds isolated from these two plants were previously investigated for efficacy against parasitic helminths. This lead information provided strong rationale for screening six major compounds isolated from *C. crispa* and *P. amabile* against the two most important genera of human helminth parasites, the nematode whipworm (*Trichuris*) and the platyhelminth blood fluke (*Schistosoma*). Other minor compounds were not studied here due to limited quantities isolated at the time of our phytochemical analysis. We used the xWORM technique^16^ that monitors helminth motility in real time using xCELLigence to investigate their anthelmintic activities and further demonstrated the promising efficacy of bergapten and isomyristicin using scanning electron microscopy (SEM) techniques.

## Results

### Preparation of phytochemicals for the study

In this study, compounds isolated from two different Bhutanese medicinal plants, *Corydalis crispa* and *Pleurospermum amabile,* were investigated for their anthelmintic activities. Focusing on alkaloids, the MeOH extract of *C. crispa* was sequentially fractionated and extracted using different solvents of increasing polarities, which finally yielded 1.34 g of crude total alkaloids. This crude alkaloid extract was purified using fractional crystallization and separation techniques as detailed in the Materials and Methods section. Through repeated separation by flash column chromatography and pre-coated silica plates, a total of nine isoquinoline alkaloids were isolated and characterised from *C. crispa*^14^ using Infrared (IR) Spectroscopy, Mass Spectrometry (ESI-MS, HR-EI-MS), Gas Chromatography Mass Spectrometry (GCMS), and Nuclear Magnetic Resonance (NMR-^1^H, ^13^C, gCOSY, gNOESY, TOCSY, gHSQC and gHMBC). Similarly, the crude MeOH extract of *P. amabile* was subjected to acid-base fractionation and repeated separation processes using the same techniques described above for *C. crispa*, which finally yielded four phenylpropanoids and six furanocoumarins^15^,^17^.

**Figure 1:**
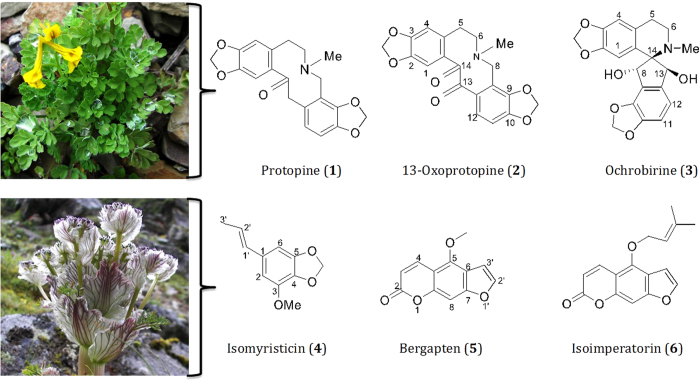
Structures of the compounds (1–6) isolated and screened for their anthelmintic activities. (A) Protopine (**1**), 13-Oxoprotopine (**2**) and ochrobirine (**3**) were isolated from *Corydalis crispa*^14^. (B) Isomyristicin (**4**), bergapten (**5**) and isoimperatorin (**6**) were isolated from *Pleurospermum amabile*^15^.

Out of 19 phytochemicals isolated in total from these two plants, six major compounds (**1–6**) were selected (Fig. 1) and screened them for their anthelmintic activities using the xWORM technique. The quantities of other minor compounds that we have isolated from these two plants were insufficient for carrying out any form of anthelmintic analyses. The selected compounds were: protopine (**1**), 13-oxoprotopine (**2**), ochrobirine (**3**), isomyristicin (**4**), bergapten (**5**) and isoimperatorin (**6**). Their structures are drawn in Fig. 1. While compounds **1–3** were isolated from *C. crispa*, compounds **4–6** were isolated from *P. amabile*.

### Trematocidal effects of compounds 1–6 against *Schistosoma mansoni* adult flukes

**Figure 2:**
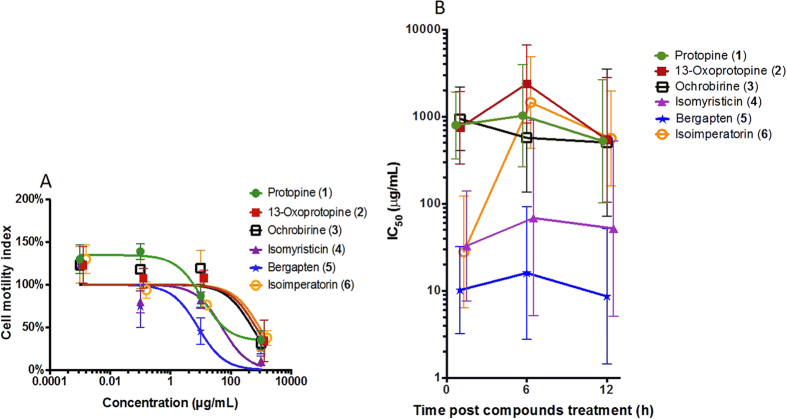
Anti-schistosome activities of six compounds (1–6) against adult *Schistosoma mansoni* determined using the xWORM technique. (**A**) Motility/mobility index dose response curves of worms 12 h after addition of test compounds at different doses (0.1**–**1000 μg/mL). (**B**) 50% inhibitory concentration (IC_50_) curves over time. Error bars represent 95% confidence intervals of the nonlinear curve fit. The curves were marginally shifted on the x-axis to aid viewing. These figures represent the data from three independent experiments.

Seven weeks post-infection, adult flukes were perfused from the mesenteries of mice and transferred immediately to Basch medium (10% fetal bovine serum and 1 × penicillin/streptomycin) for culturing at 37 °C with 5% CO_2_. After an overnight incubation at these conditions, the parasites were transferred to E-plates for drug treatments and their motility/mortality were monitored using the xWORM technique^16^. Among the six compounds (**1–6**, Fig. 1) tested here, none of the three compounds (**1–3**) isolated from *C. crispa* exhibited any substantial anti-*Schistosoma* effects. However, two compounds **–** isomyristicin (**4**) and bergapten (**5**), which were isolated from *P. amabile*, showed significant dose-dependent activity against *S. mansoni* adult flukes (Fig. 2A) with IC_50_ values of 52.0 μg/mL and 8.6 μg/mL, respectively (calculated at 12 h post addition of compounds). While the highest doses (1000 μg /mL) of both compounds killed flukes within 12 h, the lower doses (0.1**–**10 μg/mL) took longer to kill flukes as reflected by higher motility index values (Fig. 2A). Of the compounds assessed, bergapten exhibited significantly greater anti-schistosome activity at all time points with IC_50_ values of 10.2 μg/mL (1 h), 16.0 μg/mL (6 h) and 8.6 μg/mL (12 h) (Fig. 2B).

### Effects of isomyristicin and bergapten against schistosomula of *S. mansoni*

**Figure 3:**
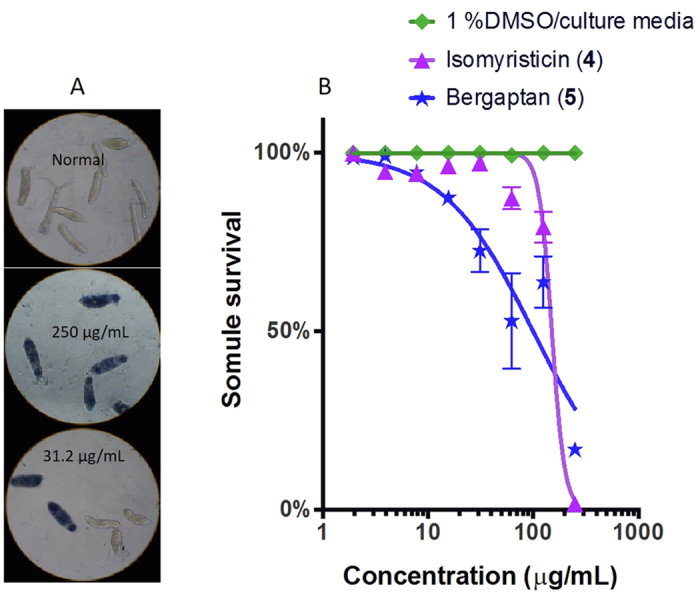
Effect of isomyristicin and bergapten on the survival of *S. mansoni* schistosomula. (**A**) Trypan blue stained images (20×) showing 100% lethality to schistosomula (250 μg/mL compound concentrations) and first signs of lethal effects observed at 31.2 μg/mL of the two compounds tested. Deep blue staining signifies a dead parasite. (**B**) The effect of the two compounds on schistosomula survival at different concentrations (250–1.95 μg/mL). The data was generated from triplicate samples obtained from two independent studies.

Praziquantel is effective against adult stage schistosomes but not against the intra-mammalian larval stage of *S. mansoni* – the schistosomulum. Since isomyristicin and bergapten showed significant anti-schistosome effects against adult flukes, we tested them against the schistosomulum stage. Schistosomula were generated by mechanical transformation of cercariae as described by Peak *et al.*^18^ and 100 of them were placed in each well of a 96 well plate containing culture media (100 μL). These schistosomula were co-cultured in the presence of the two compounds and were assessed for their survival using Trypan blue exclusion (Fig. 3A). Isomyristicin and bergapten showed lethal effects at a lowest dilution of 60 μg/mL and 16 μg/mL, respectively, achieving 85**–**100% killing (Fig. 3A). A dose response curve of schistosomula survival showed that bergapten with an IC_50_ of 100.3 μg/mL was marginally more potent than isomyristicin with an IC_50_ of 150.5 μg/mL (Fig. 3B). Schistosomula treated with 1% DMSO in culture media (as solvent control) showed no signs of toxicity or death (100% survival) as measured by Trypan blue exclusion.

### SEM analysis of *S. mansoni* treated with isomyristicin and bergapten

**Figure 4:**
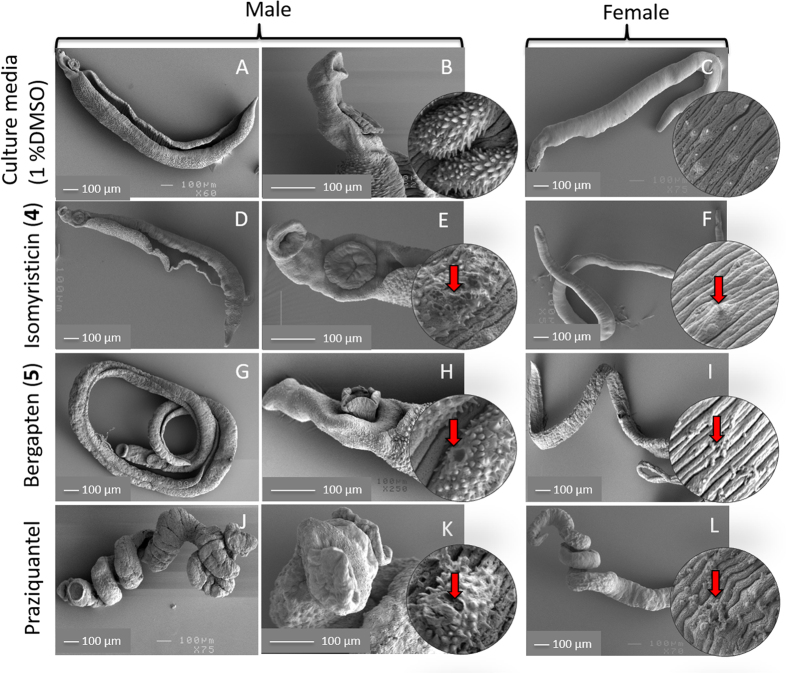
Scanning electron micrographs showing the surface morphology of *S. mansoni* treated with solvent alone, isomyristicin, bergapten and praziquantel. The SEM photos represent different groups treated with the lowest dose concentration of 4 μg/mL (all inset photos are 1 μm). (**A–C**) Worms cultured in solvent alone (1% DMSO/culture media) displayed a relaxed (uncoiled) and healthy physical appearance. (**D–F**) Isomyristicin treated parasites displayed a partially damaged physical appearance including erosion of tubercles (**E**), loss of spines and cracks (marked with red arrow) in the dorsal surface of the tegument (E, inset photo), and damage to the sensory papillae in the female tegument (F, inset photo). (**G–I**) Bergapten-treated parasites displayed a coiled appearance with similar damage to the worm teguments including erosion of tubercles (**H**) and loss of spines and cracks (marked with red arrow) on the dorsal surface of the tegument (H, inset photo), and damage to the sensory papillae in the female tegument (I, inset photo). Similar patterns but with extensive morphological changes were seen in the praziquantel-treated parasites (**J–L**).

Based on the best *in vitro* anthelmintic activity exhibited by isomyristicin and bergapten, we further investigated the effects of these compounds on the morphology of adult *S. mansoni* using SEM. The effect of praziquantel (used as positive control here) on the *S. mansoni* tegument has been assessed using SEM^19^. The SEM samples were prepared in triplicates by dividing the 24 well plates into three groups. We observed that isomyristicin, bergapten and praziquantel affected the morphology of adult worms in a dose dependent manner. The representative SEM photos of different treatment groups (all groups treated with 4 μg/mL doses) shown in Fig. 4 demonstrate the observed physiological and tegumental changes. Worms cultured in media only (with 1% DMSO in culture media as vehicle control) displayed normal physical appearance (Fig. 4A) with numerous healthy tubercles and well-formed spines in males (Fig. 4B), and clearly defined surface grooves with sensory papillae in females (Fig. 4C). On the other hand, the male *S. mansoni* that were treated with isomyristicin, while not displaying a coiled appearance (Fig. 4D), did show signs of eroded tubercles and loss of spines and formation of cracks (marked with red arrow) in the dorsal surface of the tegument (Fig. 4E).

The female worms treated with the same compound exhibited partially coiled physical appearances, and at higher SEM magnification the damage to the sensory papillae in the female tegument (Fig. 4F) was visible. Bergapten-treated parasites displayed more extensive physical and morphological changes (Fig. 4G–I). Both male and female worms showed a coiled appearance (Fig. 4G). Male worms suffered from disfigurement of oral and ventral suckers (Fig. 4H), erosion of tubercles (Fig. 4H, inset photo), loss of spines and formation of cracks/holes in the dorsal surface of the tegument (Fig. 4H, inset photo). The female worms exhibited erosion of the tegument and sensory papillae (Fig. 4I, inset photo). Similarly, praziquantel-treated parasites displayed far more extensive morphological changes (Fig. 4J–L). The degree of coiling (Fig. 4J) and the tegumental damage caused by praziquantel were visually apparent, as was that caused by bergapten and isomyristicin, which suggests that these compounds acted to damage the tegument in similar fashion.

### Nematocidal effects of compounds 1–6 against *Trichuris muris* adult whipworms

**Figure 5:**
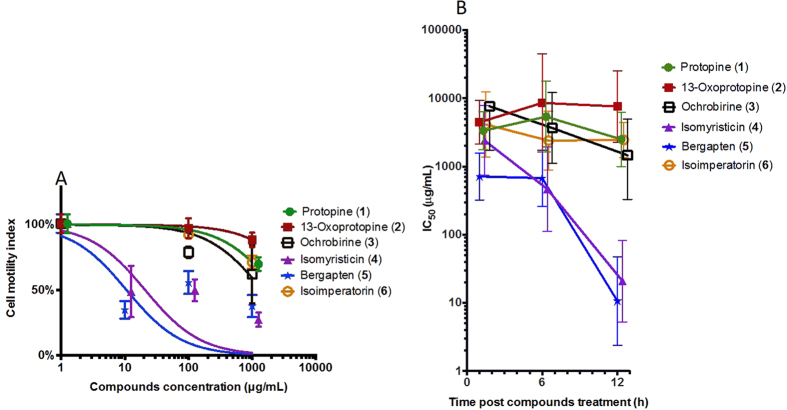
Anti-Trichuris effects of compounds (1–6) against *T. muris* determined using the xWORM technique. (**A**) Effects of different concentrations of compounds **1–6** were assessed on adult worm motility. 50% inhibitory concentration (IC_50_) values were determined at the 12 h time point. (**B**) Combined IC_50_ values of these six compounds calculated for three different doses at 1, 6, and 12 h time points. Error bars represent 95% confidence intervals of the nonlinear curve fit. The curves were marginally shifted on the x-axis to aid viewing. These figures represent the data from three independent experiments.

After four weeks, adult worms were harvested from the caecum of the mice, washed with PBS/2 × antibiotic/antimycotic (AA), resuspended in culture medium (100 μL of RPMI containing 10% foetal calf serum and AA), transferred four worms each in the E-Plates and then incubated overnight at 37 °C with 5% CO_2_. These worms were co-cultured with compounds **1–6 **at same conditions and their motility/mortality were assessed using the xWORM technique^16^. Of the six compounds (**1–6**, Fig. 1) tested, isomyristicin and bergapten again showed the best dose-dependent anti-Trichuris activity with IC_50_ values of 20.9 μg/mL and 10.6 μg/mL, respectively **–** calculated on cell motility index at the 12 h time point (Fig. 5A). The IC_50_ values of the six compounds tested were obtained using xWORM and calculated at 1 h, 6 h and 12 h time points (Fig. 5B). At 1 h post-treatment intervals, isomyristicin-treated worms appeared to be active even at the highest dose administered. However, by 6 h post-treatment, worm motility rapidly declined with an IC_50_ value similar to that of bergapten (Fig. 5B). Bergapten exhibited the best IC_50_ values (8.6**–**16.0 μg/mL) at 1 h and 12 h time points in comparison to other compounds tested.

### SEM analysis of *T. muris* treated with isomyristicin and bergapten

**Figure 6:**
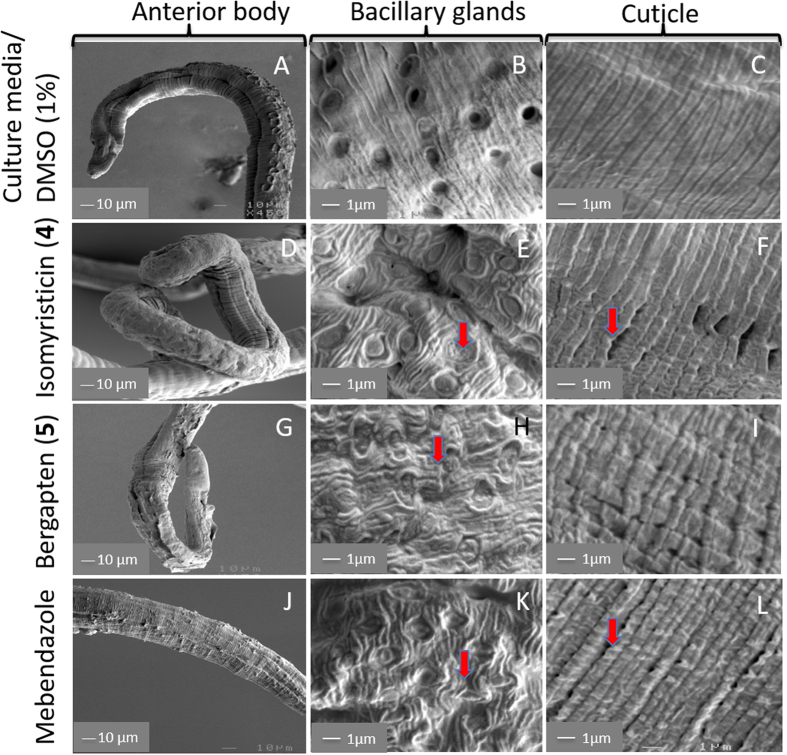
Scanning electron micrographs showing the surface morphology of *T. muris* treated with solvent alone, isomyristicin, bergapten and mebendazole. The SEM photos represent different groups treated with the lowest dose concentration of 4 μg/mL. (**A–C**) Worms treated with solvent alone (1% DMSO/culture media) displayed an unremarkable phenotype with intact bacillary bands, glands and cuticle. Worms treated with isomyristicin (**D–F**), bergapten (**G–I**) and mebendazole (**J–L**) exhibited swelling, undulation and loosening of the cuticle surrounding the bacillary glands and cuticular fissures. The red arrows show the affected regions when treated with the compounds and positive control drug.

Based on the anti-Trichuris activity of isomyristicin and bergapten (determined using xWORM), we analysed the effect of drug treatment on the morphology of *T. muris* using SEM. *T. muris* (mixed sexes) treated with culture media (1% DMSO) alone showed normal anterior body appearance (Fig. 6A) with well defined bacillary glands (Fig. 6B) and a smooth cuticle with knitted parallel segmental joins (Fig. 6C). On the other hand, the worms treated with isomyristicin (Fig. 6D–F) exhibited partial damage to the morphology of worms including swelling, loosening and destruction of bacillary glands and the cuticle. Bergapten (Fig. 6G–I) affected worm morphology in a similar way. Mebendazole-treated worms (Fig. 6J–L) (positive control) showed a higher degree of bacillary gland damage and cuticular fissures compared to the two test samples.

## Discussion

New drugs can be developed synthetically. However, the natural products, especially those compounds derived from medicinal plants including quinine and artemisinin, continue to save the lives of millions of people worldwide. Despite this proven record, medicinal plants have been relatively under-explored as a source of anthelmintic compounds. We have recently reported the anti-parasitic activities of the crude extracts and a small number of isolated compounds from the Bhutanese medicinal plants, *C. crispa* and *P. amabile*, against *Trypanosoma brucei rhodesiense* and the multi-drug resistant strain (K1CB1) of *Plasmodium falciparum*^12^,^14^,^15^. Encouraged by this report and also the traditional uses of the plants as anthelmintics^12^,^13^, we have further investigated six major compounds (out of 19 compounds isolated in total) against two distinct phyla of helminth parasites, the soil-transmitted nematode *T. muris* and the platyhelminth *S. mansoni*. We found that two of them – isomyristicin and bergapten that were isolated from *P. amabile –* have dual anthelmintic properties.

Isomyristicin and bergapten exhibited dose-dependent lethal effects against *S. mansoni* with IC_50_ values of 52.0 μg/mL and 8.6 μg/mL, respectively – which were calculated on cell motility index at the 12 h time point. At the lowest dilution of 60 μg/mL and 16 μg/mL – respectively, both compounds achieved 85**–**100% killing of schistosomula **–** the developmental stage to which praziquantel is ineffective. Isomyristicin and bergapten also demonstrated anti-*Trichuris* activity with IC_50_ values of 20.9 μg/mL and 10.6 μg/mL, respectively. Since mebendazole and albendazole are still effective in controlling the infective embryonic stage of whipworm, we did not see a need to screen them here. The ultrastructural analysis of worms treated with these two compounds revealed similar patterns of surface damage to those induced by praziquantel and mebendazole^20^,^21^,^22^,^23^. The surface damage inflicted by these two compounds resulted in coiled and shrivelled phenotypes with surface erosions at the time of death. In *T. muris*, the test compounds induced physical damage mainly around the anterior bacillary band and glands, which are responsible for excretion of digestive enzymes, pre-digestion and nutrient uptake^24^,^25^,^26^,^27^. In *S. mansoni,* both compounds damaged spines giving rise to a pitted appearance with holes/pores around the tubercles.

We expected that the broad-spectrum properties of these two compounds could be linked to their cytotoxicity profiles. However, our previous cytotoxicity evaluation^15^ showed that both compounds had negligible toxicity profiles. Interestingly, myristicin (parent chemical from which isomyristicin is derived) and bergapten are both components of vegetables/herbs/fruits consumed by humans on a daily basis. For example, parsley, celery, lemons, figs, carrots, grape juice, Earl grey tea, nutmeg, and dill have all been reported to contain these two phytochemicals^28^,^29^,^30^,^31^,^32^,^33^, albeit at very low concentrations. For example, the quantity of bergapten that can be ingested during a regular meal containing parsley is approximately 0.5**–**0.8 mg^33^. Thus, the likelihood of an anthelmintic benefit from dietary intake is low. Our readings of the published literature revealed little bioactivity information on isomyristicin, but bergapten has been subjected to a number of biological studies. Bergapten is reported as an effective molluscidal agent against *Biomphalaria glabrata* (wrongly reported as *B. boissi* by Schonberg and Latif in 1954)^34^ **–** an intermediate host of *S. mansoni* and is neither caustic nor irritating, as is the case with many synthetic molluscicides. Bergapten is also a known photomutagenic and phototoxic agent and is applied in sunscreens and in tanning cosmetic products to stimulate skin pigmentation^35^. It has been clinically studied in photochemotherapy of psoriasis, where patients treated with 1.2**–**1.6 mg/kg oral dosing experienced therapeutic benefits without side effects^36^. It has been also proposed as an active molecule to counteract survival and growth of breast hormone-responsive tumors possibly due to inhibition of cytochrome activities^30^,^37^. While these therapeutic properties of bergapten are encouraging, it has also been reported as a selective axolemmal potassium channel blocker^38^ prompting a need for further substantiatsion of its therapeutic value versus toxicity index using appropriate *in vivo* models.

Finding new anthelmintic drug lead compounds, both from natural and synthetic sources, has become crucial since there are only limited anthelmintic drugs available to combat schistosomiasis and soil-transmitted helminth infections, which together affect more than one billion people worldwide^1^,^2^,^3^. The poor cure rates described for both praziquantel and albendazole and the development of resistance to most anthelmintics used to treat livestock^39^ is a concern given the sole reliance on mass drug administration programs for human NTDs. Moreover, efforts to develop vaccines against parasitic platyhelminths and nematodes have thus far been unsuccessful, precipitating an urgent need to find arrays of new anthelmintic drug lead molecules that can be developed into more effective anthelmintics. New anti-parasitic drugs require excellent safety and therapeutic profiles, should exhibit broad spectrum activity against different types of infections, and also display significant activity against different developmental stages of parasites^40^. Therefore, finding a broad-spectrum drug that could treat multiple diseases or multiple life stages is desirable, especially in developing countries with limited resources. Isomyristicin and bergapten met these criteria in that they have anti-fluke and anti-whipworm properties. In addition, these compounds were effective at killing schistosomula, the stage of *S. mansoni* where the existing frontline drug praziquantel is ineffective. This anti-schistosomulum activity of the two compounds is interesting and warrants in-depth therapeutic analysis. Effectively controlling the early stages of infection in the human (host) prior to the onset of egg laying could prevent further damage to the lung and liver, thereby preventing the maturation of larvae into adult flukes that lodge in the portal and mesenteric vasculature.

In summary, despite their anthelmintic activity being significantly better than the untreated control group, the therapeutic effects of isomyristicin and bergapten were markedly weaker than praziquantel and mebendazole. However, unlike praziquantel or mebendazole, isomyristicin and bergapten showed widespread anti-parasitic properties – especially against the infective schistosomulum stage of *S. mansoni*. Both compounds met the criteria of the ‘Lipinski rule of 5’^41^,^42^, which predicts the drug-likeliness of a compound based on the bioavailability or membrane permeability of a chemical to an organism. They are small molecules with molecular weights less than 500 atomic mass units and have large log P values of 2**–**3, which enhance the compound permeability into the cell membrane. All these chemical and biological properties of isomyristicin and bergapten highlight their potential as lead scaffolds upon which to develop newly derivatized anthelmintic drugs. With further medicinal chemistry optimisation, including derivitization, drug structure-activity-relationship and detailed cytotoxicity studies, these two compounds offer promise as natural product anthelmintics against various life stages of multiple phylogenetically distant parasites - blood flukes and whipworms -, which cause some of the most crippling and debilitating human tropical diseases.

## Materials and Methods

### Plant materials and preparation of compounds for anthelmintic assay

The whole part of wild *Corydalis crispa* (Fumariaceae) and the aerial part of wild *Pleurospermum amabile* (Umbelliferae) were collected from alpine Himalayan mountains (altitude range of 3600**–**4800 meters above sea level) of Lingzhi in Bhutan in June**–**August 2009. They were assigned herbarium voucher specimen number 78 and 29, respectively; and were deposited at Menjong Sorig Pharmaceuticals (previously called Pharmaceutical and Research Unit), Ministry of Health in Bhutan. The air-dried plant materials (2 kg each) were chopped into small pieces and were repeatedly extracted with methanol (AR/HPLC grade, 5~ 3 L over 48 h) to obtain crude methanol extract of *C. crispa* (90 g) and *P. amabile* (190 g).

The isolation of alkaloids from the crude MeOH extract of *C. crispa* was performed as described previously^14^. The MeOH extract was acidified with HCl (5%) and then subjected to sequential fractionation and extraction using hexane (5 × 60 mL) and CH_2_Cl_2_ (5 × 60 mL). The remaining acidified aqueous solution was basified (pH 9**–**11) with NH_4_OH solution, then extracted with CHCl_3_ (5 × 60 mL). The CHCl_3_ extract was washed with H_2_O, dried with Na_2_SO_4_ and the solvent was evaporated under reduced pressure to yield a crude CHCl_3_ alkaloid extract (1.34 g). Focusing on alkaloids, the crude basic CHCl_3_ extract was subjected to the fractional crystallization and repeated separation and purification processes using flash column chromatography packed with Merck Kieselgel 60 PF254 and the pre-coated silica plates (0.2 mm silica thickness, Merck). Nine alkaloids were isolated from *C. crispa*^14^.

The isolation of phenylpropanoids and furanocoumarins from the crude MeOH extract of *P. amabile* was performed as described previously^15^. The crude extract was dissolved in MeOH/water (1:9) and fractionated first with petroleum spirit to remove fats and other neutral compounds. The remaining aqueous portion was acidified with HCl (5%) and fractionated with CH_2_Cl_2_ to remove acidic compounds. The aqueous component was again basified with NH_4_OH at pH 9**–**12 and then fractionated with CH_2_Cl_2_ to generate the basic CH_2_Cl_2_ extract**–**from which four phenylpropanoids and six furanocoumarins were isolated^15^,^17^ using column and thin layer chromatographies as specified above.

These natural products isolation protocols used UV light (short wavelength of 254 nm, long wavelength of 366 nm) and ceric ammonium molybdate (CAM) for visualization and detection of the compounds on TLC plates. Infrared (IR) Spectroscopy, Mass Spectrometry (ESI-MS, HR-EI-MS), Gas Chromatography Mass Spectrometry (GCMS), and Nuclear Magnetic Resonance (NMR-^1^H, ^13^C, gCOSY, gNOESY, TOCSY, gHSQC and gHMBC) were used for characterizing and identifying the isolated compounds. Out of 19 compounds isolated from these two medicinal plants^14^,^15^,^17^, we have selected six major compounds: protopine (**1**), 13-oxoprotopine (**2**), ochrobirine (**3**), isomyristicin (**4**), bergapten (**5**) and isoimperatorin (**6**), for screening their anthelmintic activities. The stock solutions of the six test compounds were prepared by initially dissolving 1 mg of weighed samples in 10–20 μL of DMSO and then subsequently diluting them with 980**–**990 μL of relevant culture media to make the stock concentrations of 1 mg/mL. While culturing the worms with 1 mg/mL dose concentrations, we transferred 20 μL of stock solutions into E-plate wells containing parasites in 180 μL of fresh media (without DMSO). The final volume in each well was 200 μL (20 μL test solutions + 180 μL of fresh media). To achieve other dose concentrations (0.1**–**200 μg/mL), we pre-diluted the stock solutions to make that required concentration and then added to the E-plates as performed in the initial dosing experiment. These were performed in triplicate with a final in-well compound concentration ranging from 0.1**–**1000 μg/mL. Each replicate contained solvent control (1% DMSO in culture media). For schistosomula drug assays, stock solutions were diluted in culture media with two-fold dilutions to obtain in-well dose concentrations of 2**–**1000 μg/mL.

### Preparation of *S. mansoni* adult flukes

*S. mansoni* cercariae were shed from infected *Biomphalaria glabrata* snails (Biomedical Research Institute, MD, USA) by exposure to light at 26 °C for 2 h. BALB/c mice (12–14 weeks old male) were infected with cercariae (~120 cercariae/mouse) by abdominal penetration as reported previously^43^. Seven weeks post-infection, adult flukes were perfused from the mesenteries of mice and transferred immediately to Basch medium (10% fetal bovine serum and 1 × penicillin/streptomycin) for culturing at 37 °C with 5% CO_2_ as previously reported^44^. After an overnight incubation at 37 °C with 5% CO_2_ parasites were transferred to E-plates (ACEA Bioscience Inc., USA) for motility assessment using the xWORM technique or SEM, described below.

### Schistosomula preparation

Schistosomula were generated by mechanical transformation of cercariae as described by Peak *et al.*^18^ Schistosomula (100 μL volume containing ~100 schistosomula) were placed in 96 well plates containing culture media (100 μL) and divided into triplicate wells for each compound. Parasites were treated with the test compounds at various in-well concentrations of 2**–**1000 μg/mL achieved through two-fold dilution. Parasites were cultured at 37 °C with 5% CO_2_ for 12**–**40 h and stained with trypan blue solution to assess viability after treatment. The live and dead flukes in each well were counted manually under a light microscope and 50% inhibitory concentration (IC_50_) values were calculated for each compound.

### Preparation of adult ***Trichuris muris***

Genetically susceptible mice were orally infected with approximately 200 *T. muris* eggs. After 4 weeks the mice were sacrificed and adult worms were harvested from the caecum. The worms were washed with PBS/2 × antibiotic/antimycotic (AA), resuspended in culture medium (100 μL of RPMI containing 10% foetal calf serum and AA). After an overnight incubation at 37 °C with 5% CO_2_ parasites were transferred to E-plates (ACEA Bioscience Inc., USA) for motility assessment using the xWORM technique or SEM, described below.

### xWORM technique

The xWORM technique was employed using an xCELLigence SP system (ACEA Biosciences Inc., USA) as described previously^16^,^45^. All experiments were carried out as per the manufacturer’s instructions with 15 sec read intervals using the real time cell assay (RTCA) software (ACEA Biosciences Inc., USA). Separated (single) and paired adult *S. mansoni* flukes per well (mixed gender) were used in the xWORM assay (two independent experiments), and four adult *T. muris* of mixed gender were placed in each well of the 96 well E-plates containing a final volume of 200 μl of culture medium. All assays were conducted in triplicate. Inter-well spaces were filled with 100 μL of culture medium or PBS to prevent evaporation. The E-plates containing worms were cultured overnight at 37 °C with 5% CO_2_ to obtain a baseline motility reading. The worms were then treated with prepared concentrations of the test compounds and the motility of the worms was monitored for 12–40 h.

### IC_50_ calculations and statistical analyses

As described previously^16^,^45^, we determined the IC_50_ values of test compounds based on the motility index for adult worms. The motility index was calculated as the standard deviation (SD) over 800 data points (i.e. 4 readings per min for 200 min) of the cell index (CI) difference from the rolling average over 20 data points (10 proceeding and preceding CI values - 5 min total). While 100% motility was determined from the average motility index of the untreated wells, 0% motility was determined from a media only well (no worms present). The 100% motility was converted from the motility index averaged over 100 data points (25 min). This figure was then used in GraphPad Prism 6.0 to calculate and compare IC_50_ values. We used a log (test compound concentration) vs normalised response (100–0%) formula, with variable slope when data were sufficient or set −1 hill slope when data was limited, and outliers were automatically removed (with default ROUT coefficient used: Q = 1%). The IC_50_ values for each dose concentration were calculated at 1, 6, and 12 h post-treatment of the worms with the test compounds. Compounds with IC_50_ values of higher than 100 μg/mL were considered ineffective in this study. When data were sufficient to use the variable slope statistical analysis, the Hill Slope and the Log IC_50_ value were together compared for significant differences using an extra sum-of squares F-test of GraphPad Prism 6.0.

### Scanning electron microscopy

Adult worms were treated with prepared concentrations of test compounds. To determine how the compounds impacted on the morphology of adult worms, we used modified SEM methods detailed in Stepek *et al.*^22^. For *S. mansoni*, we used the methods described by Edwards *et al.*^21^. Mebendazole was used as a positive control for anti-*Trichuris* activity and praziquantel was used as a positive control for anti-*Schistosoma* activity. Treated worms were prepared for SEM as follows: a) fixed in gluteraldehyde (3%) in Sorensen’s buffer overnight; b) dehydrated (15 min each) with a graded ethanol series (50%, 60%, 70%, 80%, 90%, 100%), mixture of ethanol and hexamethyldisilizane (HMDS) (1:1 ratio), and then finally with pure HMDS (100%); c) dried overnight in a fume hood. Two or three dried worms from each treatment regimen were placed on an aluminum stub, sputtered with gold and then visualized using a JEOL JSM scanning electron microscope (10 kV). To assess the morphological changes, we scanned each worm from anterior to posterior and obtained digital images of the affected region using Semaphore software.

### Ethics and clearances

 All experimental work involving mice was approved by the James Cook University (JCU) animal ethics committee (Ethics approval number A2213). Mice infected with S. mansoni and T. muris were raised in cages at the JCU animal facility under normal conditions of regulated temperature (22 °C) and lighting (12 h light/dark cycle) with free access to pelleted food and water. The mice were kept for 4–7 weeks in cages in compliance with the Australian Code of Practice for the Care and Use of Animals for Scientific Purposes, 7th edition, 2007 and the Queensland Animal Care and Protection Act 2001.

## Additional Information

**How to cite this article**: Wangchuk, P. *et al.* Identification of lead chemotherapeutic agents from medicinal plants against blood flukes and whipworms. *Sci. Rep.* **6**, 32101; doi: 10.1038/srep32101 (2016).
